# Domestic

**Published:** 1840-08-01

**Authors:** 


### DOMESTIC.

*Natchez Tornado.*—We have seen several short notices of this desolating tempest by gentlemen of Natchez, from which we propose embodying some of the more remarkable facts. According to Dr. Tooley, whose account is the fullest that we have read, the morning of the fatal 7th of May was densely overcast, and very warm, with a brisk south wind which increased about noon, veering to the east. The southwestern sky at mid-day assumed a darker and more tempestuous aspect, the gloom and turbulence increasing every moment; and by forty-five minutes after 12 the storm began to be distinctly heard, the wind blowing a gale from the northeast. The roar of the tempest, which grew louder and more terrific as it advanced rapidly upon the city, was attended with incessant flashes of forked lightning. At 1.45, Dr. Tooley describes the storm-cloud as assuming "an almost pitchy darkness, curling, rushing, roaring above, below, a lurid yellow, dashing upward, and rapidly approaching, striking the Mississippi some six or seven miles below the city, spreading desolation upon each side, the western side being the centre of the annulus. At this time a blackness of darkness overspread the heavens; and when the annulus approached the city, the wind suddenly veered to the S. E. 8, attended with such crashing thunder as shook the solid earth. At 2.10 the tornado burst upon the city, dashing diagonally through it, attended with such murky darkness, roaring and crashing, that the citizens saw not, heard not, knew not the wide wasting destruction around them." The rush of the tornado over the city occupied a space of time not exceeding five minutes, and the destructive blast not more than a few seconds. At this moment the barometer fell, according to one writer, to nearly 29.

The disastrous effects of the storm are too well known to the readers of the Journal to require a lengthened description. "Natchez under the Hill," with the exception of one or two houses, was razed to the ground, and nearly every private dwelling and public edifice in the city sustained more or less injury. Hundreds of houses were unroofed, or had their gable ends or windows blown out; of three steamboats at the wharf, two were sunk, and the third, which was freighted with lead, had its upper works blown away to the water's edge; not less than sixty flat boats parted their cables, and were swamped; and three hundred human beings, it is computed, perished on the land and in the river during the few moments in which the tempest was passing. Few such storms are recorded in the history of the United States; but as hurricanes of destructive violence occur almost every year in some part of the country, it becomes a matter of something more than curious interest to ascertain the laws by which they are governed, and the mode in which they exert their tremendous force. We were informed by Dr. Cartwright, that Dr. Tooley preserved his house from all injury, even the breaking of a pane of glass, by adopting the measures which his theory of storms suggested. That theory was the *explosive* one—that, where houses are demolished by a tornado, it is in consequence of the sudden expansion of the air within, caused by the instantaneous rarefaction of the external atmosphere. Dr. Tooley observed, that as the storm approached the mercury in his barometer sunk rapidly; and he prepared for the expansion of the air in his house by raising all the windows and throwing open the doors. His house was not so well built to resist a storm as many of those in his neighbourhood which were prostrated, or sustained more or less damage, and its escape can only be accounted for by the fact, that he provided for the exit of the air which, confined, must have blown out the windows, as happened in many instances, if it had not blown down the house. A wing of Dr. Cartwright's house was blown down, but the main body of it which was of a very substantial structure, escaped with the loss of its chimneys and the bursting out of the windows.

What is the rationale of tornadoes? Is the force exerted owing to the gyratory motion of the atmosphere, or to a sudden rarefaction in some portion of it, causing a corresponding expansion of those portions immediately under it or around it? In many storms there can be no doubt, that the gyrations of the atmosphere do the mischief, as where forest trees are seen twisted off. In other cases the violent sweep of the atmosphere bears down all before it. But in Natchez the wind is said not to have been more violent than the persons who were present had often seen it when no extensive mischief was done; and this tornado, from a multitude of facts collected, seems to have been of the class in which the ruin results from explosions. The following may be cited from a great number:

1. The gardener of Dr. Cartwright had just quit his employ, and in leaving his house neglected to close the doors and windows. It escaped without injury. The gardener of a friend, living in his immediate neighbourhood, hastened when he saw the storm approaching, and succeeded in closing his doors and windows, which he had scarcely done when the house fell upon him and killed him.

2. The garret of a brick house, mentioned in the account of Dr. Tooley, being closely shut up, both ends were burst outward, and with such explosive force, that some of the bricks of the windward end were thrown upon a terrace nearly on a level with it, to a distance of not less than twenty feet, in the face of the wind.

3. A brick house on the north side of Main street had its leeward gable end blown out, the windward end remaining uninjured.

4. The windward gable end of a large house adjoining the Commercial Bank, bursted outward in the face of the storm, the leeward end escaping without injury.

5. The gable ends of a large three-story brick house on Franklin street were thrown out with great violence, in opposite directions, and one, of course, against the wind.

6. The leeward ends of two brick stores were thrown outward with violence, while the windward ends escaped. The same happened to the leeward side of a large brick house close by.

7. In the neighbourhood of the last mentioned, another brick house had the windward gable end thrown outward.

8. The desks in the Agricultural Bank, which were locked by the president as the storm commenced, were found open shortly after, with their locks bursted. In another instance, the drawer of a bureau was thrown quite out, while the bureau itself was found in its previous position.

9. The leeward walls of two front rooms of the Tremont House were thrown outward with great force, without injuring or disturbing the furniture within.

10. The gable-ends of a large brick store on Main and Pearl streets, were blown out; the roof of the fire-proof brick office of the Probate Court exploded to windward; and in a house on State street a large trap-door in the roof was bursted open, giving an outlet to the air, and saving the roof.

Hundreds of such facts, it is said by persons who have surveyed the ruins, might be adduced, showing, that where sufficient openings were not afforded to the expanding air, the roof, windows, or some other part of the house gave way, and most generally to the leeward. A writer in one of the Natchez papers pledges himself to point out to the incredulous, in a walk through the city, *five hundred explosions*—instances in which the violence done can only be explained by the outward action of the atmosphere.

We have a parallel case in the *break-bottle* experiment with the air-pump, in which a thin square bottle, hermetically sealed, is shivered into a thousand fragments, under the exhausted receiver, by the expansion of the confined air. The pressure of the atmosphere over the city was suddenly diminished nearly one-thirtieth, as was shown by the fall of the barometer, and rooms containing four thousand cubic feet of air, were thus subjected, it has been estimated, to a pressure from within of eighty-six tons more than from without. The consequence was, that the windows were blown out when the walls were strong, and the equilibrium was thus restored; and in garrets, where the air was more confined, trap-doors were blown open, or gable ends thrown out with immense force. In some cases roofs were heaved up and removed, and often, as has been shown, walls were shot out in the face of the wind. Garrets being closer were oftener exploded than other apartments which were relieved by windows and doors; and for the same reason brick houses sustained more damage than those composed of wood. And, finally, in the "explosive" theory we have an explanation of the well-authenticated fact, that where doors and windows were unclosed, leeward and windward, houses, as was strikingly the case with Dr. Tooley's, escaped all injury. Whatever, therefore, may be the *modus operandi* of hurricanes generally, the conclusion seems irresistible, that in the tornado at Natchez the demolition of buildings was occasioned by the rarefaction of the outer atmosphere, and a corresponding expansion of the air within, equalling the explosive force of gunpowder. Still, there are phenomena connected with the storm for which nothing but the supposition of "a mighty rushing wind" will account; and such a wind, in fact, is inseparable from the rarified state of the air which led to the explosions. Into the air which thus presented a comparative vacuum, the surrounding atmosphere must have rushed with great violence; and it was this wind that uprooted forest trees, raised the immense waves in the Mississippi, and forced the boats from their moorings.

The quantity of rain which fell during the passage of the tornado, according to Dr. Tooley, was only 83-100th of an inch, but holding in suspension mud and particles of leaves and other vegetable matter in such quantities as not only to darken the air, but leave a thick coating upon whatever it came in contact with.

Dr. Tooley closes his account of the tornado with a description of some curious effects produced by it upon the leaves and buds of plants: they were in a manner *seared* by it. Those which were not killed outright were crisped, and their growth suspended for ten or more days. Some very thriving grape cuttings in the garden of Dr. T. were killed, and the old vines were also stunted and injured. An arbour vitæ in his yard seemed blighted and dying; the leaves of the succulent morus multicaulis appeared for some days as if an eastern sirocco had passed over them; and fruit trees, grass, and weeds, assumed the same appearance.—*Western Journ. of Med. and Surg.*

